# Comparison of the frequencies of ENU-induced point mutations in male germ cells and inherited germline mutations in their offspring

**DOI:** 10.1186/s41021-021-00216-z

**Published:** 2021-10-09

**Authors:** Kenichi Masumura, Tomoko Ando, Naomi Toyoda-Hokaiwado, Akiko Ukai, Takehiko Nohmi, Masamitsu Honma

**Affiliations:** 1 Division of Genetics and Mutagenesis, National Institute of Health Sciences, 3-25-26 Tonomachi, Kawasaki-ku, Kawasaki-shi, Kanagawa 210-9501 Japan; 2 Division of Pathology, National Institute of Health Sciences, 3-25-26 Tonomachi, Kawasaki-ku, Kawasaki-shi, Kanagawa 210-9501 Japan

**Keywords:** *gpt* delta transgenic mouse, Mutation frequency, Whole genome sequencing, Germline mutation, *de novo* mutation

## Abstract

**Background:**

Gene mutations induced in germ cells may be transmitted to the next generation and cause adverse effects such as genetic diseases. Certain mutations may result in infertility or death in early development. Thus, the mutations may not be inheritable. However, the extent to which point mutations in male germ cells are transmitted to the next generation or eliminated during transmission is largely unknown. This study compared mutation frequencies (MFs) in sperm of *N*-ethyl-*N*-nitrosourea (ENU)-treated *gpt* delta mice and *de novo* MFs in the whole exome/genome of their offspring.

**Results:**

Male *gpt* delta mice were treated with 10, 30, and 85 mg/kg of ENU (i.p., weekly × 2) and mated with untreated females to generate offspring. We previously reported a dose-dependent increase in *de novo* MFs in the offspring estimated by whole exome sequencing (WES) (Mutat. Res., 810, 30–39, 2016). In this study, *gpt* MFs in the sperm of ENU-treated mice were estimated, and the MFs per reporter gene were converted to MFs per base pair. The inherited *de novo* MFs in the offspring (9, 26 and 133 × 10^− 8^/bp for 10, 30, and 85 mg/kg ENU-treated groups, respectively) were comparable to those of the converted *gpt* MFs in the sperm of ENU-treated fathers (6, 16, and 69 × 10^− 8^/bp). It indicated that the *gpt* MFs in the ENU-treated father’s sperm were comparable to the inherited *de novo* MFs in the offspring as estimated by WES. In addition, *de novo* MFs in the offspring of 10 mg/kg ENU-treated and control fathers were estimated by whole genome sequencing (WGS), because WES was not sufficiently sensitive to detect low background MF. The *de novo* MF in the offspring of the ENU-treated fathers was 6 × 10^− 8^/bp and significantly higher than that of the control (2 × 10^− 8^/bp). There were no significant differences in *de novo* MFs between gene-coding and non-coding regions. WGS analysis was able to detect ENU-induced characteristic *de novo* base substitutions at a low dose group.

**Conclusions:**

Despite a difference between exome/genome and exogenous reporter genes, the results indicated that ENU-induced point mutations in male germ cells could be transmitted to the next generation without severe selection.

**Supplementary Information:**

The online version contains supplementary material available at 10.1186/s41021-021-00216-z.

## Introduction

**Fig. 1:**
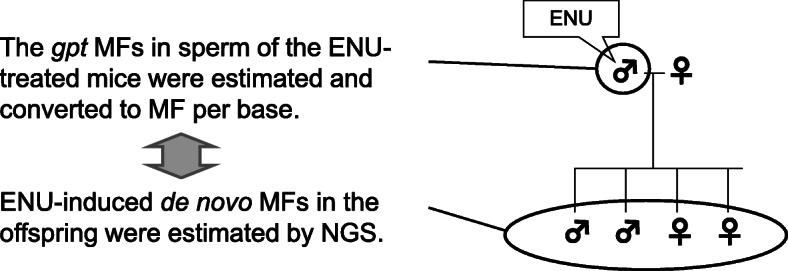
Comparison of MFs in the sperm of the ENU-treated mice and *de novo* germline MFs in the offspring

Germline mutations are a source of *de novo* mutations and genomic diversity in a population. Mutations induced in germ cells may be transmitted to the next generation and possibly result in adverse effects such as genetic diseases [1, 2]. Therefore, germ cell mutation analysis and risk evaluation for the subsequent generation is important in genetic toxicology [3]. However, some genotoxic changes in germ cells may result in infertility or death during early development; thus, mutations should be eliminated before the next generation. For example, most structural and numerical chromosomal abnormalities are unstable and cause embryonic lethality [4]. However, it is unknown to what extent point mutations in male germ cells are transmitted to the next generation. Recent advances in high-throughput sequencing technology mean we can detect *de novo* germline mutations [5–7]. *N*-ethyl-*N*-nitrosourea (ENU) is an alkylating agent that is a germ cell mutagen. ENU preferentially induces point mutations, and pre-meiotic spermatogonial stem cells are particularly sensitive to its effects [8, 9]. We previously demonstrated a dose-dependent increase of *de novo* mutations in the offspring of ENU-treated male *gpt* delta mice by whole exome sequencing (WES) analysis [10, 11]. We also observed a dose-dependent induction of mutations in the sperm of ENU-treated fathers using a *gpt* gene reporter assay. However, the *de novo* MF estimated by WES analysis could not be directly compared with the *gpt* mutant frequency in the father’s sperm because the *gpt* mutant frequency represents the frequency per reporter gene estimated by bacteria-mediated phenotypic selection [12]. This study compared the MF in ENU-treated male germ cells and *de novo* MF in the offspring. The *gpt* assay was performed in sperm, and the estimated *gpt* MFs per reporter gene was converted to MFs per base pair (Fig. 1). In the previous study, however, we could not detect *de novo* mutations in the control group because the background MF was too low to detect mutations in whole exome regions. Whole genome sequencing (WGS) can have more sequencing data than WES and have an adventage to detect smaller mutagenic response. In this study, WGS analysis was conducted to estimate *de novo* MFs in the control and the low dose ENU-treated group (10 mg/kg × 2). *De novo* MFs and mutation spectra were analyzed, and the MFs between gene-coding and non-coding regions were compared.

## Materials and methods

### Treatment of animals

Male and female *gpt* delta mice (C57BL/6J background) [12, 13] were obtained from a colony maintained at the National Institute of Health Sciences. The animal experiments are described in previously published studies [10, 11]. Briefly, nine-week-old male mice were treated with ENU (10, 30, and 85 mg/kg body weight, intraperitoneally, weekly on two occasions). The highest dose of ENU was set at 85 mg/kg, because 100 mg/kg ENU resulted in permanent infertility in a preliminary experiment. It is considered the maximum tolerated dose in the germline (data not shown). Control male mice were treated with phosphate/citrate buffer as a vehicle. Animal treatment was carried out in two separate experiments (control and 85 mg/kg ENU as experiment 1; control, 10, and 30 mg/kg ENU as experiment 2). Ten weeks after the last treatment, the mice were mated with untreated females. Five male mice were used in each group, and 10 mice were used for vehicle control. The males (28 to 30 weeks old), mated females (28 to 33 weeks old), and the offspring (5 weeks old) were euthanized. The tissues were collected and stored at − 80 °C. The cauda epididymis of the fathers was used for the mutation assay, and the livers of the parents and offspring were used for genome sequencing analysis.

### Reporter gene mutation assay

Sperm DNA was extracted as previously described [10]. Briefly, the cauda epididymis was sliced in phosphate-buffered saline, filtered, and pelleted by centrifugation. The pellet was resuspended in 1× saline sodium citrate (SSC) and 0.15 % sodium dodecyl sulfate (SDS). The lysate was centrifuged, and the sperm pellet was resuspended in 0.2× SSC, 1 % SDS, 1 M 2-mercaptoethanol, and 10 mM EDTA (pH 8.0), and then digested overnight with 0.5 mg/mL proteinase K at 37 °C. DNA was isolated by phenol/chloroform extraction, ethanol precipitation, and resuspended in TE buffer (pH 8.0).

Lambda EG10 transgenes were rescued from genomic DNA by *in vitro* packaging reactions using Transpack Packaging Extract (Agilent Technologies). The *gpt* mutation assay was performed as previously described [13]. Briefly, the rescued phages infect *Escherichia coli* YG6020, which express Cre recombinase to convert the transgene into a plasmid. The infected cells were mixed with molten soft agar and poured onto agar plates containing chloramphenicol (Cm) and 6-thioguanine (6TG). The plates were incubated at 37 °C to select colonies that harbored the plasmid carrying the mutated *gpt* gene. Infected cells were also poured onto plates containing Cm without 6TG to determine the number of rescued plasmids. The *gpt* mutant frequencies were calculated by dividing the number of 6TG-resistant colonies by the number of rescued plasmids. In order to correct for clonality, 1 to 9 *gpt* mutants from each animal were sequenced. A DNA fragment containing the *gpt* sequence was amplified by direct colony PCR and *gpt* mutations were characterized by Sanger sequencing with a sequencing primer (5′-TCTCGCGCAACCTATTTTCCC-3′). We estimated clonally corrected MF as “independent MF”. Independent *gpt* MFs were calculated by the mutant frequency × ratio of independent mutations among sequenced mutants (Supplemantary table S1). The specific *gpt* MF of each mutation type was calculated by *gpt* MF × proportion of the mutation type.

### Conversion of *gpt* MF per reporter gene to MF per nucleotide base pair

The conversion rate from *gpt* MF per reporter gene to MF per base pair was calculated as follows. In the *gpt* mutation assay, only mutations resulting in a substantial loss of enzymatic activity of guanine phsophoribosyltransferase (*gpt* gene product) can be detected as a 6TG-resistant mutant colony. The number of these “phenotypically detectable” positions and the types of *gpt* mutations were assessed (Supplementary Fig. S1). Only base substitutions were determined for simplification purposes. The *gpt* gene is 459 bp, and each nucleotide has three possible substitutions (for example, A could be substituted to G, C, and T). Thus, the total number of possible single base substitutions in the *gpt* gene is 459 ⋅ 3 = 1,377. Previously sequenced *gpt* mutations (from published and unpublished data) were mapped to the 1,377 *gpt* mutation types. A total of 3,330 single base substitutions were mapped to only 342 mutation types. In addition, we found 13 other possible mutation types that could induce the same AA changes as those detected previously. The rate of phenotypically detectable mutations in the *gpt* sequence was calculated as (342 + 13)/1,377 = 0.2578. Finally, the *gpt* MF per gene was converted to the MF per base pair using the following formula:

$${\mathrm{MF}}_{\mathrm{base}}\;=\;{\mathrm{MF}}_{\mathrm{gene}}\;/\left(459\;\times\;0.2578\right)\;=\;{\mathrm{MF}}_{\mathrm{gene}}\;\times\;0.0085\\{\mathrm{MF}}_{\mathrm{gene}}:\;gpt\;\mathrm{MF}\;\mathrm{per}\;\mathrm{reporter}\;\mathrm{gene}\\{\mathrm{MF}}_{\mathrm{base}}:\;gpt\;\mathrm{MF}\;\mathrm{per}\;\mathrm{base}\;\mathrm{pair}$$

### Whole exome/genome sequencing and estimation of *de novo* germline MF

The methods for WES analysis were described previously [11]. Briefly, each ENU-treated family and one control family were sequenced. One of the ENU-treated fathers from each dose group (ID 8 for 85 mg/kg, ID 044 for 30 mg/kg, and ID 029 for 10 mg/kg) and one control father (ID 43) were randomly selected. Each family consisted of six mice [i.e., parents (male and female) and offspring (two males and two females)]. DNA was extracted from the liver using a DNA Extractor WB Kit (Wako, Osaka, Japan). DNA samples from 24 mice were subjected to WES by Beckman Coulter Genomics (MA, USA) and data analyses by Genaris Omics Inc. (Kanagawa, Japan). The mouse exome (49.6 Mb) was captured using a SureSelect Mouse All Exon Kit (Agilent Technologies) and sequenced using a Hiseq2000 (Illumina, CA, USA) with 100-bp paired ends. Over 5 Gb of the sequenced data per animal were mapped to the exon region, resulting in approximately 100-fold redundancy. The reference sequence was C57BL/6J mouse genome NCBI Build 37 (mm9) since the SureSelect Mouse All Exon Kit was based on the same reference sequence. The sequenced reads were mapped onto the reference sequence using BWA. Duplicated reads were deleted with the Picard tool. For each animal, single nucleotide variants (SNVs) were called based on comparisons with the reference sequence using GATK. The detected SNVs were annotated by SnpEff.

*De novo* mutations in the exomes of offspring were previously identified by Trio analysis as described [11]. The SNVs were compared between the parents and offspring, and *de novo* mutations were identified as follows: (1) SNVs potentially transmitted from parents to offspring were excluded; (2) Unique SNVs found in only a single offspring (absent in the 23 other mice) were considered *de novo* mutations; (3) NGS genotype quality (GQ) scores greater than 20 (= 99 % accuracy) for a Trio (i.e., father, mother, and offspring) were selected; (4) The read depth (i.e., the number of sequenced reads that covered one nucleotide position) needed to exceed a cut-off value in a Trio. The cut-off value was 27 in the WES analysis; (5) Only mutations of which an alternate read ratio is 0.3 ≤ ratio ≤ 0.7 were selected. The alternate read ratio was defined as a ratio of the number of variants read out of the total number of reads at a sequenced position; and (6) the consensus sequence had to be homozygous and identical in the other 23 mice. The frequency of *de novo* germline mutations was calculated as follows: The number of *de novo* mutations was divided by the number of bases in the exome sorted with the same cut-off value (i.e., a minimum read depth and GQ score in a Trio).

WGS analyses were performed as follows: One of 10 mg/kg ENU-treated family (ID 029) and one control family (ID 43) were sequenced. Both families consisted of parents (male and female) and offspring (two males and two females). DNA was extracted from the liver using a DNA Extractor WB Kit (Wako). WGS and data analyses were conducted by Genebay Inc. (Kanagawa, Japan). DNA samples from 12 mice were sequenced using Hiseq X (Illumina) with 150-bp paired ends. Over 90 Gb of the sequenced data per animal were mapped to the whole genome in a size of 3 Gb, resulting in approximately 30-fold redundancy. The reference sequence was the C57BL/6J mouse genome GRCm38 (mm10). The sequenced reads were mapped using BWA. Duplicate reads were deleted by the Picard tool. For each animal, single nucleotide variants (SNVs) were called using GATK. SnpEff annotated the detected SNVs.

*De novo* mutations in the whole genome of offspring were identified by Trio analysis. For WGS analysis, *de novo* mutations were identified as follows: (1) SNVs that were potentially transmitted from parents to offspring were excluded; (2) Unique SNVs found in only a single offspring (absent in the 11 other mice) were selected; (3) GQ scores greater than 20 for a Trio were selected; (4) The read depth had to exceed a cut-off value in a Trio, and the cut-off value was 26 in the WGS analysis; (5) Alternate read ratio was 0.3 ≤ ratio ≤ 0.7; (6) Consensus sequence homozygous and identical to the other 11 mice; (7) False indels and base substitutions caused by mapping errors were checked using IGV and excluded; and (8) Clustered mutations possibly caused by sequencing errors and/or contaminated reads were excluded. The frequency of *de novo* germline mutations was calculated as follows: The number of *de novo* mutations was divided by the number of bases in the whole genome sorted with the same cut-off value and GQ score in a Trio. In addition, the *de novo* MFs for gene-coding and non-coding regions were also estimated by the same methods, except for different cut-off values (24 for gene-coding regions, 26 for non-coding regions).

### Statistical analysis

MFs in each dose group are presented with standard deviation (SD). Comparisons of MFs between ENU-treated groups versus vehicle control were analyzed by Tukey’s test or Steel’s test. For WGS analyses, a comparison of the MFs between the ENU-treated group versus the control was analyzed by a Student’s t-test.

## Results

### Comparison of *gpt* MFs in the sperm of ENU-treated mice and *de novo* germline MFs in the offspring by WES

**Fig. 2:**
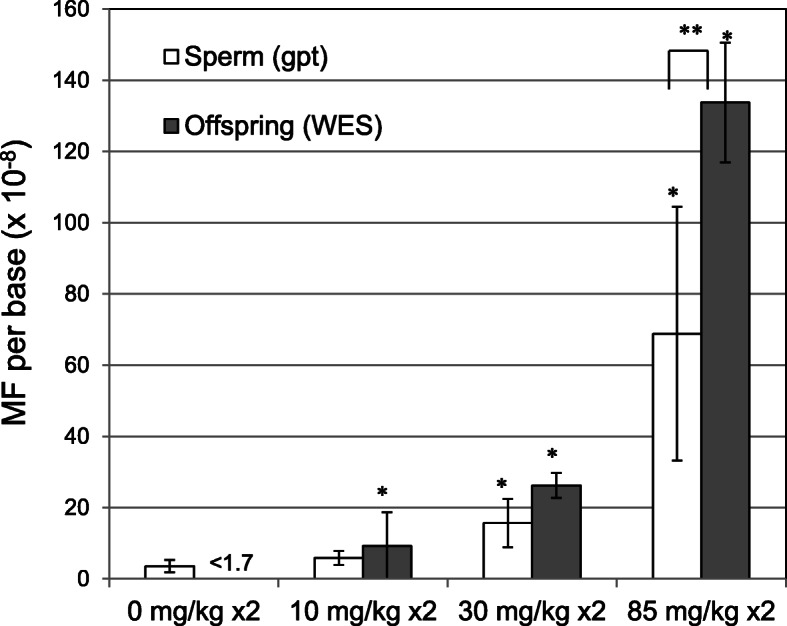
The *gpt* MFs in sperm of the ENU-treated father and *de novo* germline MFs in whole exome in the offspring. For *gpt* MFs in sperm, each of ENU-treated group had 5 mice, and control group had 10 mice. For *de novo* MF in offspring, each group had 4 siblings. Error bars represents SD. *: P < 0.05, significantly different from control (Steel test). **: p < 0.01, significantly different between sperm (*gpt*) and offspring (WES) (Tukey test)

ENU-treated male mice were mated with untreated females and the offspring were obtained as previously reported [10, 11]. *De novo* MFs in the offspring, calculated by WES, were reported in the previous study [11]. The *de novo* MFs per 10^8^ bases for 10, 30, and 85 mg/kg ENU-treated groups were 9.3 ± 9.4 SD, 26.2 ± 3.5 SD, and 134 ± 17 SD. For the control, the MF was < 1.7 because no mutations were detected in the sorted exome sequence. Dose-dependency and a statistically significant increase occurred in the ENU-treated groups. In order to investigate the frequency of point mutations per nucleotide base in germ cells of the ENU-treated father, a *gpt* assay was performed using sperm DNA isolated from ENU-treated and control mice. Estimated *gpt* MFs were converted to MF per nucleotide base. The calculated *gpt* MFs are shown in Fig. 2 (and Supplementary Table S1). The *gpt* MF per 10^8^ bases for the control, 10, 30, and 85 mg/kg ENU-treated groups were 3.6 ± 1.5 SD, 5.9 ± 2.0 SD, 15.6 ± 6.8 SD, and 69 ± 36 SD, respectively. There was a dose-dependent increase in the *gpt* MF. A statistically significant increase was observed in the 30 and 85 mg/kg ENU-treated groups.

**Fig. 3:**
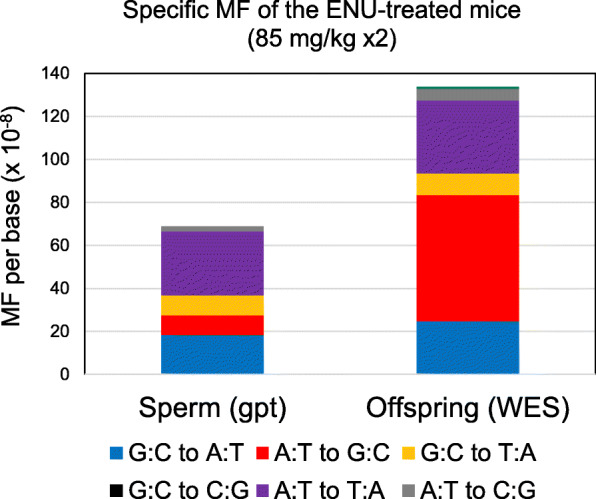
Mutation Spectra of the *gpt* mutations in sperm of the ENU-treated mice and *de novo* germline mutations in whole exome in the offspring

The *gpt* MFs in the ENU-treated father’s sperm were comparable to the inherited *de novo* MFs in the offspring as estimated by WES. At the highest dose, the *de novo* MF in the offspring was significantly higher than the MF in the sperm, although it was less than a 2-fold difference. The mutation spectra of the *gpt* mutations in sperm of the 85 mg/kg ENU-treated father and *de novo* germline mutations in the offspring are shown in Fig. 3. G:C to A:T transitions and A:T to T:A transversions were predominantly detected in both father’s sperm (*gpt* assay) and offspring (WES), and the specific MFs were similar. In contrast, A:T to G:C transitions were detected more frequently by WES than the *gpt* assay.

### *De novo* MF in the offspring of the ENU-treated fathers by WGS

**Fig. 4:**
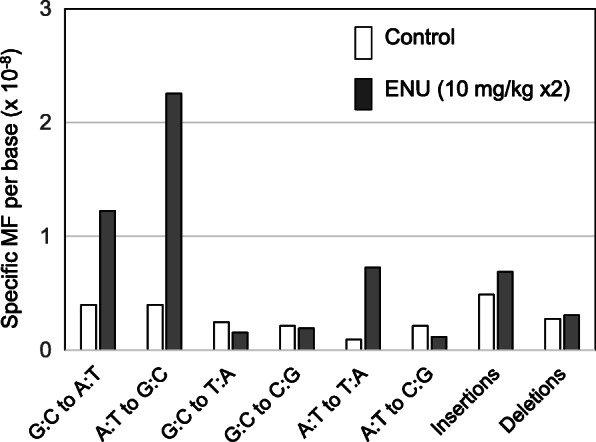
Mutation spectra of the ENU-induced *de novo* germline mutations detected by WGS in the offspring of the ENU-treated and control fathers

**Fig. 5:**
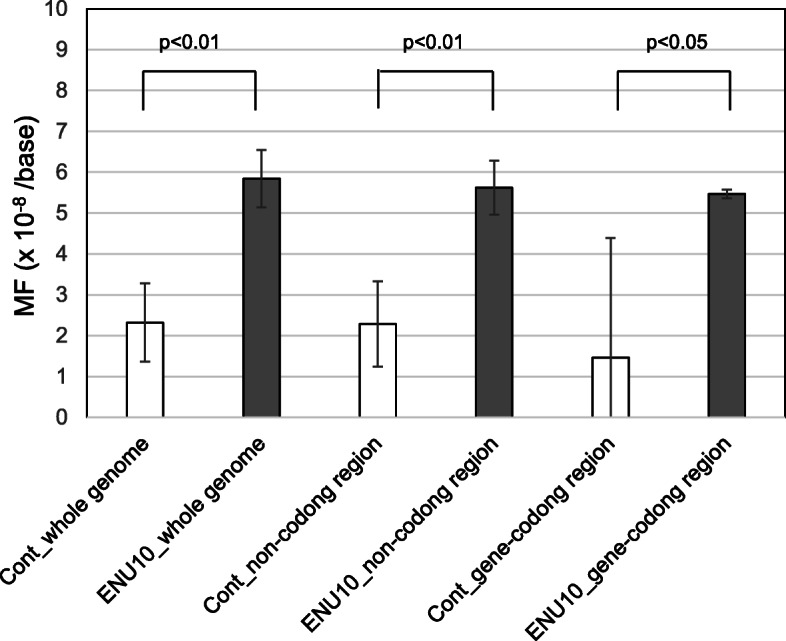
*De novo* germline MFs in gene-coding and non-coding regions. *De novo* germline MFs detected by WGS in control and 10 mg/kg ENU-treated group were calculated for each of whole genome, gene-coding region, and non-coding region

To estimate *de novo* MF in the offspring of the control and low-dose ENU-treated group, WGS analysis was conducted for the same families used for WES. For the WGS analysis, the animal ID 529 exhibited a higher number of mutations than those in siblings. The data analysis suggested that contaminated reads caused many sequencing errors; therefore, ID 529 was excluded as an outlier for calculating the average MF. *De novo* germline MFs per 10^8^ bases in the offspring of the control and 10 mg/kg ENU-treated father were 2.3 ± 1.0 SD and 5.7 ± 0.7 SD, respectively (Table 1). The *de novo* MF in the offspring of the ENU-treated father was 2.5-fold significantly higher compared with that of the control. The mutation spectra are shown in Fig. 4 (and Supplementary Table S2). G:C to A:T and A:T to G:C transitions and A:T to T:A transversions, were preferentially induced by ENU. These are similar types to the ENU-induced mutations observed by WES analysis. *De novo* germline MFs detected by WGS were also calculated for each gene-coding and non-coding region (Fig. 5). No significant difference was observed in *de novo* MFs between these regions. No mutational hotspots were observed in the control and ENU-induced mutations (Supplementary Table S3). Regarding spontaneous *de novo* mutations, G:C to A:T, A:T to G:C, and small indels were the major types. The small indels detected by WGS were mostly observed in short tandem repeats (Supplementary Table S4), and they were 2 ~ 23 repeats of the sequences of 1 ~ 12 bps.

**Table 1:** De novo germline MFs detected by WGS in the offspring of ENU-treated male mice

Offspring ID		Father ID	Mother ID	No. of bases sequenced	No. of mutations	De novo MF (×10^− 8^/base)	Average		SD	
Control										
501	male	43	50	759,410,873	10	1.3				
502	male	43	50	765,916,678	14	1.8				
503	female	43	50	878,853,903	23	2.6				
504	female	43	50	824,878,986	29	3.5	**2.3**	**±**	**1.0**	
ENU (10 mg/kg × 2)										
523	male	29	52	884,658,265	45	5.1				
524	male	29	52	888,192,187	48	5.4				
528	female	29	52	849,121,370	55	6.5				
529	female	29	52	924,310,341	248	26.8	10.9	±	10.6	
							(S529 is excluded.)			
							**5.7**	**±**	**0.7**	*

* *P* < 0.01, t-test

## Discussion

The mice were treated with ENU and mated with untreated females 10 weeks after the last treatment to ensure the germ cells were exposed at the spermatogonial stem cell stage. MFs in the sperm were estimated using a reporter gene mutation assay (*gpt* assay). To calculate the conversion rate from *gpt* MF per reporter gene to the MF per base, phenotypically detectable mutation types of the *gpt* gene were identified. Of the 1,377 theoretically possible single base substitutions in *gpt*, only 24.8 % (342/1377) were detected in a cohort of 3,330 previously identified mutations by *gpt* assay. This indicates that there are limited mutation types and positions in the *gpt* gene that can be phenotypically detected. In order to simplify the calculation, indels were excluded from the analysis. Although indels could inactivate a gene, it is difficult to define all possible indels. In addition, the majority of spontaneous mutations and ENU-induced mutations are base substitutions. Therefore, the calculated conversion formula is a rough estimate and useful for the *gpt* gene. Different reporter genes may have different conversion rates depending on their sequence context. Converted *gpt* MFs in the sperm of fathers were estimated to be 3.6, 5.9, 16, and 69 × 10^− 8^/bp for 0, 10, 30, and 85 mg/kg ENU-treated groups, respectively. The *de novo* MFs in the offspring were < 1.7, 9.3, 26, and 133 × 10^− 8^/bp for the 0, 10, 30, and 85 mg/kg ENU-treated groups, respectively [11]. At the highest dose (85 mg/kg), the *de novo* MF in the offspring was significantly higher compared with the MF in the sperm of fathers. The mutation spectra in the sperm and the *de novo* germline mutations in the 85 mg/kg ENU-treated group indicated that G:C to A:T and A:T to T:A were the predominant mutations in the ENU-treated group (Fig. 3). These are common characteristics of ENU-induced mutations as reported in various transgenic rodent gene mutation assays [10, 14–18]. ENU induces a variety of DNA adducts of which *O*^4^-ethylthymine, *O*^2^-ethylthymine, and *O*^6^-ethylguanine are considered responsible for base substitutions [8, 19–23]. Interestingly, A:T to G:C transitions were detected more frequently by WES in the offspring than the *gpt* assay in the sperm of the fathers. This mutation type mainly contributed to the difference between the *gpt* MF in sperm versus the *de novo* MF in the offspring. This may be caused by a difference in the method of mutation detection. Revollo et al. reported mutation spectra of single T-cell clones of ENU-treated F344 rats analyzed by WGS [24]. ENU-induced somatic mutation spectrum in T-cells showed approximately 40 % A:T to G:C transitions, followed by A:T to T:A and G:C to A:T. This is remarkably similar to the ENU-induced *de novo* germline mutation spectrum analyzed by WES in this study. This suggests that the ENU-induced mutation spectrum obtained by the NGS-based study appears to be quite similar between somatic and germline mutations. On the other hand, exogeneous reporter genes may be relatively insensitive to A:T to G:C transitions [10]. There is no clear explanation that sequence context and/or detection methods may cause detection bias. For example, hot spots of *gpt* spontaneous mutations are observed at position 64, 110, and 115 in the *gpt* gene. Those are G:C bps at CpG sites, and may contribute to higher sensitivity to mutations at G:C bps. These results suggest that the majority of ENU-induced point mutations in the father’s germ cells could be transmitted to the genome of the next generation without severe selection during development.

For the WES analysis, *de novo* mutations were not identified in the control group because of the small exome size (~ 50 Mb), which is insufficient to detect extremely low MFs [11]. In order to estimate the background mutation frequency, *de novo* MFs in the offspring of control and 10 mg/kg ENU-treated fathers were analyzed by WGS. WGS analysis estimated that *de novo* MF per 10^8^ bases in the offspring of 10 mg/kg ENU-treated fathers was 5.7± 0.7 SD and 2.5-fold higher compared with the control (2.3± 1.0 SD) (Table 1). This was a comparable range to that estimated by WES (9.3 ± 9.4 SD for 10 mg/kg ENU-treated group and < 1.7 for control), but more precisely by WGS analysis. Animal ID 529 was excluded as an outlier because many sequencing errors caused by contaminated sequence reads were observed. Minor DNA contamination from other species could affect NGS analysis in the estimation of the *de novo* MF. The mutation spectrum analyzed by WGS showed that G:C to A:T, A:T to G:C, and A:T to T:A transitions were induced by ENU (Fig. 4), which was the same characteristics as detected by WES. The *de novo* MFs were also estimated for the gene-coding and non-coding regions by WGS (Fig. 5). The results suggested no significant differences in sensitivity of the ENU-induced germline mutations between gene-coding and non-coding regions under these experimental conditions. For the spontaneous *de novo* mutations detected by WGS, G:C to A:T, A:T to G:C, and small indels were the major types observed. Interestingly, the indels detected by WGS were mostly observed in short tandem repeats (Supplementary Table S4). These indels may indicate microsatellite instability in the genome and/or sequencing errors in tandem repeat sequences. To confirm that these indels are real, Sanger sequencing or long-read NGS analysis may be necessary. The germline mutation rate in humans was estimated to be approximately 1 × 10^− 8^ per base per generation [5]. The germline MFs in C57BL/6 mice was estimated to be 0.54 × 10^− 8^ per base [25]. The control MF, estimated as 2.3± 1.0 SD per 10^8^ bp by WGS in this study, was slightly higher than that of the MFs. This suggests that the identified mutations in this study may contain some sequencing errors, because the mutations were not confirmed by Sanger sequencing or amplicon sequencing. The estimation of very low mutation frequency may be affected by amount of read data and detection method of *de novo* mutations in WGS analysis. Further studies are necessary to determine the *in vivo* spontaneous mutation frequency and spectrum in more detail. WGS is a more powerful technique to detect extremely low MF compared with WES. Direct sequencing analysis could be a useful tool to investigate inherited germline mutations induced by environmental mutagens.

## Conclusions

This study compared MFs in sperm of ENU-treated male mice and inherited MFs in their offspring’s by WES. The MFs in male germ cells were increased by ENU in a dose-dependent manner and the MFs were comparable to that of the inherited *de novo* MFs in the offspring. This suggests that point mutations induced in male germ cells may be transmitted to the next generation without severe exclusion during fertilization and development. The WGS was more appropriate than the WES for estimation of low MFs. *De novo* mutations were induced by ENU in both coding and non-coding regions at similar frequencies.

## Supplementary Information

**Additional file 1: Supplementary Figure S1.** Position and type of “phenotypically detectable” point mutations in the *gpt* gene by 6TG selection.

**Additional file 2: Supplementary Table S1.** The *gpt* MFs in the sperm of ENU-treated *gpt* delta mice. **Supplementaly Table S2.** Mutation spectra in the offspring of control and ENU-treated mice (WGS). **Supplementaly Table S3.** List of mutations in the offspring of control and ENU-treated mice (WGS). **Supplementaly Table S4.** Indels detected by WGS in the offspring of ENU-treated fathers.

## Data Availability

Data generated or analyzed during this study are included in this published article and supplementary information files.

## Abbreviations

6TG: 6-thioguanine
BWA: Burrows-Wheeler Aligner
Cm: Chloramphenicol
ENU: *N*-ethyl-*N*-nitrosourea
MF: Mutation frequency
SD: Standard deviation
WES: Whole exome sequencing
WGS: Whole genome sequencing
