# Supplementary material for: Comparison of the frequencies of ENU-induced point mutations in male germ cells and inherited germline mutations in their offspring
Source: Genes Environ. 2021 Oct 9;43:43. doi: 10.1186/s41021-021-00216-z (PMC8501628; doi:10.1186/s41021-021-00216-z)
Supplement: Supplementary file 1 — Additional file 1: Supplementary Figure S1. Position and type of “phenotypically detectable” point mutations in the gpt gene by 6TG selection. [file 41021_2021_216_MOESM1_ESM.docx]

**Supplementary fig. S1. Position and type of “phenotypically detectable” point mutations in the gpt gene by 6TG selection**

Name : gpt gene

Sequence Position: 1 - 459

Translation Position: 1 - 459

----------10--------20--------30--------40--------50--------60

atgagcgaaaaatacatcgtcacctgggacatgttgcagatccatgcacgtaaactcgca

MetSerGluLysTyrIleValThrTrpAspMetLeuGlnIleHisAlaArgLysLeuAla

Aaaaaaaaaaaaaaaaaaaaaaaaaaaaaaaaaaaaaaaaaaaaaaaaaaaaaaaaaaaa

gggggggggggggggggggggggggggggggggggggggggggggggggggggggggggg

cccccccccccccccccccccccccccccccccccccccccccccccccccccccccccc

tttttttttttttttttttttttttttttttttttttttttttttttttttttttttttt

----------70--------80--------90-------100-------110-------120

agccgactgatgccttctgaacaatggaaaggcattattgccgtaagccgtggcggtctg

SerArgLeuMetProSerGluGlnTrpLysGlyIleIleAlaValSerArgGlyGlyLeu

aaaaaaaaaaaaaaaaaaaaaaaaaaaaaaaaaaaaaaaaaaaaaaaaaaaaaaaaaaaa

gggggggggggggggggggggggggggggggggggggggggggggggggggggggggggg

cccccccccccccccccccccccccccccccccccccccccccccccccccccccccccc

tttttttttttttttttttttttttttttttttttttttttttttttttttttttttttt

---------130-------140-------150-------160-------170-------180

gtaccgggtgcgttactggcgcgtgaactgggtattcgtcatgtcgataccgtttgtatt

ValProGlyAlaLeuLeuAlaArgGluLeuGlyIleArgHisValAspThrValCysIle

aaaaaaaaaaaaaaaaaaaaaaaaaaaaaaaaaaaaaaaaaaaaaaaaaaaaaaaaaaaa

gggggggggggggggggggggggggggggggggggggggggggggggggggggggggggg

cccccccccccccccccccccccccccccccccccccccccccccccccccccccccccc

tttttttttttttttttttttttttttttttttttttttttttttttttttttttttttt

---------190-------200-------210-------220-------230-------240

tccagctacgatcacgacaaccagcgcgagcttaaagtgctgaaacgcgcagaaggcgat

SerSerTyrAspHisAspAsnGlnArgGluLeuLysValLeuLysArgAlaGluGlyAsp

aaaaaaaaaaaaaaaaaaaaaaaaaaaaaaaaaaaaaaaaaaaaaaaaaaaaaaaaaaaa

gggggggggggggggggggggggggggggggggggggggggggggggggggggggggggg

cccccccccccccccccccccccccccccccccccccccccccccccccccccccccccc

tttttttttttttttttttttttttttttttttttttttttttttttttttttttttttt

---------250-------260-------270-------280-------290-------300

ggcgaaggcttcatcgttattgatgacctggtggataccggtggtactgcggttgcgatt

GlyGluGlyPheIleValIleAspAspLeuValAspThrGlyGlyThrAlaValAlaIle

aaaaaaaaaaaaaaaaaaaaaaaaaaaaaaaaaaaaaaaaaaaaaaaaaaaaaaaaaaaa

gggggggggggggggggggggggggggggggggggggggggggggggggggggggggggg

cccccccccccccccccccccccccccccccccccccccccccccccccccccccccccc

tttttttttttttttttttttttttttttttttttttttttttttttttttttttttttt

---------310-------320-------330-------340-------350-------360

cgtgaaatgtatccaaaagcgcactttgtcaccatcttcgcaaaaccggctggtcgtccg

ArgGluMetTyrProLysAlaHisPheValThrIlePheAlaLysProAlaGlyArgPro

aaaaaaaaaaaaaaaaaaaaaaaaaaaaaaaaaaaaaaaaaaaaaaaaaaaaaaaaaaaa

gggggggggggggggggggggggggggggggggggggggggggggggggggggggggggg

cccccccccccccccccccccccccccccccccccccccccccccccccccccccccccc

tttttttttttttttttttttttttttttttttttttttttttttttttttttttttttt

---------370-------380-------390-------400-------410-------420

ctggttgatgactatgttgttgatatcccgcaagatacctggattgaacagccgtgggat

LeuValAspAspTyrValValAspIleProGlnAspThrTrpIleGluGlnProTrpAsp

aaaaaaaaaaaaaaaaaaaaaaaaaaaaaaaaaaaaaaaaaaaaaaaaaaaaaaaaaaaa

gggggggggggggggggggggggggggggggggggggggggggggggggggggggggggg

cccccccccccccccccccccccccccccccccccccccccccccccccccccccccccc

tttttttttttttttttttttttttttttttttttttttttttttttttttttttttttt

---------430-------440-------450-------460

atgggcgtcgtattcgtcccgccaatctccggtcgctaa

MetGlyValValPheValProProIleSerGlyArg***

aaaaaaaaaaaaaaaaaaaaaaaaaaaaaaaaaaaaaaa

ggggggggggggggggggggggggggggggggggggggg

ccccccccccccccccccccccccccccccccccccccc

ttttttttttttttttttttttttttttttttttttttt

Total number of position and type of base substitutions in gpt gene are 459 bps x 3 = 1,377

black [459]: Original base

gray [321]: Synonymous mutation (no change in amino acid)

green [342]: Observed gpt mutations (previously sequenced 3330 single base changes)

yellow [13]: Nonsynonymous mutation that causes the same amino acid changes of the observed gpt mutations

Synonymous mutation rate of gpt gene: 321/(459*3)=0.23

Nonsynonymous mutation rate of gpt gene: (459*3-321)/(459*3)=0.77

Detectable mutation rate of gpt gene: (342+13)/(459*3)=0.2578

MF_gene_: gpt mutation frequency (x 10^-6/reporter gene)

MF_base_: gpt mutation frequency (x 10^-8/base pair)

**MF_gene_/(459 x 0.2578) x 100 = MF_base_**

**MF_gene_ x 0.0085 = MF_base_**
